# Supplementary material for: Mapping the existing body of knowledge on new and repurposed TB vaccine implementation: A scoping review
Source: PLOS Glob Public Health. 2024 Aug 22;4(8):e0002885. doi: 10.1371/journal.pgph.0002885 (PMC11340902; doi:10.1371/journal.pgph.0002885)
Supplement: S1 Text — (DOCX) [file pgph.0002885.s001.docx]

# S1 Text – Search Strings

| **Database** | **Concept** | **Search string** | **Filters** |
| --- | --- | --- | --- |
| PubMed | #1 tuberculosis | “Tuberculosis”[MeSH Terms] |  |
|  | #2 Tuberculosis vaccination | “Tuberculosis vaccines” [MeSH Terms] OR “tb vaccin*”[Title/Abstract] OR “tuberculosis vaccin*”[Title/Abstract] |  |
|  | #3 Vaccine preparedness | “Immunization Programs”[MeSH Terms] OR “Vaccination Refusal”[MeSH Terms] OR “Vaccination Coverage”[MeSH Terms] OR “Vaccination Hesitancy”[MeSH Terms] OR “implement*”[Title/Abstract] OR “strateg*”[Title/Abstract] OR “barrier*”[Title/Abstract] OR “enabler*”[Title/Abstract] OR “factor*”[Title/Abstract] OR “health system*”[Title/Abstract] OR “prepare*”[Title/Abstract] OR “readiness”[Title/Abstract] OR “roll out”[Title/Abstract] OR “impact”[Title/Abstract] OR “effect*”[Title/Abstract] OR “intro*”[Title/Abstract] OR “uptake”[Title/Abstract] OR “accept*”[Title/Abstract] OR “availab*”[Title/Abstract] OR “access*”[Title/Abstract] OR “feasib*”[Title/Abstract] OR “human resources*”[Title/Abstract] OR “capacity*”[Title/Abstract] OR “coordinat*”[Title/Abstract] OR “training*”[Title/Abstract] OR “cost*”[Title/Abstract] OR “financ*”[Title/Abstract] OR “govern*”[Title/Abstract] OR “regulat*”[Title/Abstract] OR “service deliver*”[Title/Abstract] OR “scale up*”[Title/Abstract] OR “scalib*”[Title/Abstract] OR “hesitan*” [Title/Abstract] OR “facilitat*” [Title/Abstract] |  |
|  | Combined with exclusion | #1 AND #2 AND #3 AND “Humans” [Mesh] NOT “Clinical Study” [Publication type] NOT “Infant” [Mesh] NOT “Child, Preschool” [Mesh] NOT “Immunity” [Mesh] | Year: 2013-2023 |
| Medrvix | Tuberculosis vaccin | for term “tuberculosis vaccin” and abstract or title “adolescent adult” (match any words) and posted between "1^st^ of Jan 2013 and 30^th^ of Apr, 2023" | Year: 2013-2023 |
| Plos Global public health | Vaccine and tuberculosis | ((abstract:vaccin) AND abstract:tuberculosis) | Year: 2013-2023 |
