# Supplementary material for: Mapping the existing body of knowledge on new and repurposed TB vaccine implementation: A scoping review
Source: PLOS Glob Public Health. 2024 Aug 22;4(8):e0002885. doi: 10.1371/journal.pgph.0002885 (PMC11340902; doi:10.1371/journal.pgph.0002885)
Supplement: S1 Table — (DOCX) [file pgph.0002885.s002.docx]

S1 Table. Number of included studies per country, vaccine readiness focus, vaccine candidate and profile, and vaccine endpoint.

|  | **LMIC’s/high burden countries** | **China** | **India** | **South Africa** | **Cambodia** | **Indonesia** |
| --- | --- | --- | --- | --- | --- | --- |
| **Nr. studies** | **7** | **6** | **9** | **7** | **1** | **1** |
| **Vaccine readiness focus** | | | | | | |
| Epidemiological | **4** [17–19,21] | **4** [22,24,28,30] | **6** [24,26–28,31,32] | **4** [23–25,33]; | **1** [29] | **1** [27] |
| Costing, cost-effectiveness, economic | **4** [20,21,34,35] | **2** [28,37]; | **5** [27,28,32,36,37] | **3** [23,33,36] | - | 1[27] |
| Acceptability | - | **1** [38] | **1** [38] | **1** [38] | - | - |
| Implementation feasibility | - | **1** [38] | **2** [26,38] | **4** [23,25,38,39] | - | - |
| **Vaccine candidate or profile** | | | | | | |
| Hypothetical^1^ | **6** [17,19–21,34,35] | **3** [22,28,30] | **4** [26,28,31,32] | **2** [25,39] | **1** [29] | **-** |
| M72/AS01 | **1** [18] | **3** [24,28,38] | **6**[24,27,32,36–38] | **4** [23,24,36,38] | **-** | **1** [27] |
| BCG revaccination | - | **2** [24,38] | **3** [24,32,38] | **4** [24,33,38,39], | **-** | **-** |
| **Endpoint hypothetical vaccines** | | | | | | |
| PoD^2^ | **6** [17,19–21,34,35] | **2** [22,28] | **3** [26,28,31] | **2** [25,39] | **1** [29] | **-** |
| PoI3 | **-** | **2** [28,30] | **3** [28,31,32] | **1** [39] | **1** [29] | **-** |
| PoR^4^ | **-** | **-** | **-** | **-** | **-** | **-** |
