# Supplementary material for: Mapping the existing body of knowledge on new and repurposed TB vaccine implementation: A scoping review
Source: PLOS Glob Public Health. 2024 Aug 22;4(8):e0002885. doi: 10.1371/journal.pgph.0002885 (PMC11340902; doi:10.1371/journal.pgph.0002885)
Supplement: S2 Table — (DOCX) [file pgph.0002885.s003.docx]

S2 Table. Overview characteristics included articles.

| First author, year, | Type of literature | Type of study | country | Type of vaccine | Target population | Implementation strategy | Health impact | Econ-omic impact | Acceptability | Feasibility |
| --- | --- | --- | --- | --- | --- | --- | --- | --- | --- | --- |
| Pelzer, 2021 | white | Qual-itative | China, India, South Africa | BCG revax & MS72 /AS01E | various; adolescents, general population, elderly, healthcare workers, high risk contacts, socially vulnerable groups, biological high-risk groups, | mass campaigns, school program, routine immunization program, workplace, health visitations |  |  | x | x |
| Harris, 2019 | white | Modelling | China | Hypothetical^1^ vaccine (PoD^2^) | elderly & adolescents | yearly routine vaccination; catch up campaigns for specific age groups | x |  |  |  |
| Hatherill, 2016 | white | Supp-lement | South Africa | Hypothetical^1^ vaccine (PoD^2^/PoI^3^) and BCG revax | Healthcare workers | not specific |  |  |  | x |
| Harris, 2022 | white | Modelling | India, South Africa | MS72/AS01E | adolescents | routine vaccination |  | x |  |  |
| Shrestha, 2017 | white | Modelling | South Africa | Hypothetical^1^ vaccine (PoD^2^) | miners and mine-labor-sending community | not specific | x |  |  | x |
| Dye, 2013 | white | Modelling | South Africa | BCG revax | adolescents (teenagers) | not specific | x | x |  |  |
| Arinaminpathy, 2022 | Pre-print | Modelling | 24 high burden countries^4^ | Hypothetical^1^ vaccine (PoD^2^) | general population | not specific | x |  |  |  |
| Liu, 2017 | white | Modelling | China | Hypothetical^1^ vaccine (PoI^3^) | general population | mixed vaccination: constant vaccination among new and repurposed pulse vaccination strategy among all other age groups through government agencies and companies | x |  |  |  |
| Fu, 2021 | white | Modelling | high RR-TB^5^ incidence countries^6^ | MS72/AS01E | adults, adolescents | routine vaccination and catch-up campaigns amongst those over 15-years old | x |  |  |  |
| Clark, 2023a | preprint | modelling | India | BCG revax, MS72 /AS01E, hypothetical^1^ vaccine (PoI^3^) | general adults/adolescents | combination of routine vaccination, mass campaigns and a repeat campaign | x | x |  |  |
| Portnoy, 2023 | white | modelling | 105 LMIC^7^ | Hypothetical^1^ vaccine (PoD^2^) | general adults/adolescents | routine vaccination and a onetime vaccination campaign |  | x |  |  |
| Weerasuriya, 2021a | white | modelling | China, India | Hypothetical^1^ vaccine (PoD^2^ & PoI^3^) | early adolescents | annual routine vaccination, co-delivered with HPV vaccine; mass campaigns | x | x |  |  |
| Clark, 2023b | white | modelling | 105LMICs^7^ | Hypothetical^1^ vaccine (PoD^2^) | adults/adolescents | routine vaccination; mass campaign | x |  |  |  |
| Portnoy, 2022a | preprint | modelling | 105LMICs^7^ | Hypothetical^1^ vaccine (PoD^2^) | general adults/adolescents | an initial mass vaccination campaign followed by routine vaccinations |  | x |  |  |
| Portnoy, 2022b | preprint | modelling | 105LMICs^7^ | Hypothetical^1^ vaccine (PoD^2^) | general adults/adolescents | Routine vaccination plus a one-time vaccination campaign |  | x |  |  |
| Harris, 2020 | white | modelling | China, India, South Africa | BCG revax & MS72 /AS01E | general adults and adolescents | routine vaccination and mass campaigns | x |  |  |  |
| Weerasuriya, 2021b | white | modelling | China, India | MS72/AS01E | early adolescents | mass vaccination |  | x |  |  |
| Awad, 2020 | white | modelling | India | Hypothetical^1^ vaccine (PoD^2^ & PoI^3^) | people with diabetes | not specific | x |  |  |  |
| Renardy, 2019 | white | modelling | Cambodia | Hypothetical^1^ vaccine (PoD^2^ & PoI^3^) | general adults/adolescents | not specific | x |  |  |  |
| Knight, 2014 | white | modelling | LMICs^7^ | Hypothetical^1^ vaccine (PoD^2^) | general adults/adolescents | school and mass vaccination programs | x | x |  |  |
| Shrestha, 2016 | white | modelling | India | Hypothetical^1^ vaccine (PoD^2^) | general adults/adolescents | yearly routine vaccination and 10y-periodic revaccination | x |  |  | x |
| Silva, 2021 | preprint | modelling | India, Indonesia | MS72/AS01E | general adults | not specific | x | x |  |  |
| Jayawardana, 2022 | white | qualitative +modelling study | South Africa | MS72/AS01E | adults/adolescents + PLHIV adults | adults/adolescents: 1. mass + routine and 2. 2x mass. PLHIV adults: 1. mass & routine, 2. 2xmassadults | x | x |  | x |

### 1.Hypothetical vaccine= hypothetical vaccines often align with the WHO Preferred Product Characteristics (PPC)9 and do not adhere to the specific vaccine candidate characteristics currently in the pipeline, 2.PoD= prevention of disease, 3.PoI=prevention of infection, 4. High burden countries= Afghanistan, Bangladesh, Burma, Cambodia, India, Indonesia, Pakistan, Philippines, Vietnam, DR Congo, Ethiopia, Kenya, Malawi, Mozambique, Nigeria, South Africa, Tanzania, Uganda, Zambia, Zimbabwe, Kyrgyz Republic, Tajikistan, Ukraine, Uzbekistan,, 5. RR-TB= Rifampicin resistant TB, 6.high RR-TB incidence countries= India, Pakistan, Indonesia, Philippines, Myanmar, Bangladesh, Ethiopia, Russia Federation, Ukraine, south Africa, Mozambique, DR Congo, Zimbabwe, Nigeria, Thailand, Angola, Kenya, China, Vietnam, DPR Korea, Kazakhstan, Uzbekistan, Somalia, Peru, Kyrgyzstan, Papua New Guinea, Tajikistan, Belarus, Republic of Moldova, Azerbaijan, 7.LMICs= Low-income countries (LIC), and lower and upper middle-income countries (LMIC). LICs are countries with a GNI per capita of $1,135 or less and LMICs with a GNI per capita between $1,136 and $4,465. Upper middle-income economies are those with a GNI per capita between $4,466 and $13,845. GNI per capita may differ per year ([World Bank Country and Lending Groups – World Bank Data Help Desk](https://datahelpdesk.worldbank.org/knowledgebase/articles/906519-world-bank-country-and-lending-groups)),
