# Supplementary material for: Mapping the existing body of knowledge on new and repurposed TB vaccine implementation: A scoping review
Source: PLOS Glob Public Health. 2024 Aug 22;4(8):e0002885. doi: 10.1371/journal.pgph.0002885 (PMC11340902; doi:10.1371/journal.pgph.0002885)
Supplement: S3 Table — (DOCX) [file pgph.0002885.s004.docx]

S3 Table. Study characteristics and main measurement outcomes from epidemiological impact studies by country.

|  | **LMICs^1^** | **India** | **South Africa** | **China** | **Indonesia** | **Cambodia** |
| --- | --- | --- | --- | --- | --- | --- |
| **Nr. studies** | 4 | 6 | 4 | 4 | 1 | 1 |
| **Vaccine type** | | | | | | |
| BCG | 0 | 2 | 2 | 1 | 0 | 0 |
| M72/AS01E | 1 | 3 | 2 | 1 | 1 | 0 |
| Hypothetical^2^ vaccine | 3 | 4 | 1 | 3 | 0 | 1 |
| **Endpoint hypothetical vaccines** | | | | | | |
| PoI^3^ | 0 | 3 |  | 2 | 0 | 1 |
| PoD^4^ | 3 | 3 | 1 | 2 | 0 | 1 |
| PoR^5^ | 0 | 0 | 0 | 0 | 0 | 0 |
| **Target population** | | | | | | |
| Adults and adolescents | 4 | 5 | 2 | 2 | 1 | 1 |
| Other | 0 | 1^6^ | 3^7^ | 2^8^ | 0 | 0 |
| **Measurement of outcomes** | | | | | | |
| Incidence | 5^9^ | 5^6^ | 4 | 4^6^ | 0 | 1 |
| Mortality | 3 | 4^6^ | 2 | 3^6^ | 1 | 0 |

### 1.LMICs= Low-income countries (LIC), and lower and upper middle-income countries (LMIC). LICs are countries with a GNI per capita of $1,135 or less and LMICs with a GNI per capita between $1,136 and $4,465. Upper middle-income economies are those with a GNI per capita between $4,466 and $13,845. GNI per capita may differ per year ([World Bank Country and Lending Groups – World Bank Data Help Desk](https://datahelpdesk.worldbank.org/knowledgebase/articles/906519-world-bank-country-and-lending-groups)), 2.hypothetical vaccine=hypothetical vaccines often align with the WHO Preferred Product Characteristics (PPC)9 and do not adhere to the specific vaccine candidate characteristics currently in the pipeline, 3. PoI=prevention of infection, 4. PoD=prevention of disease, 5. PoR=prevention of recurrence, 6. People with diabetes mellitus, 7. Miners and labor sending community, PLHIV, adolescents only, 8. General population, adolescents & elderly, 9. Of which 1 focuses on rifampicin resistant TB.
