# Supplementary material for: Mapping the existing body of knowledge on new and repurposed TB vaccine implementation: A scoping review
Source: PLOS Glob Public Health. 2024 Aug 22;4(8):e0002885. doi: 10.1371/journal.pgph.0002885 (PMC11340902; doi:10.1371/journal.pgph.0002885)
Supplement: S4 Table — (DOCX) [file pgph.0002885.s005.docx]

S4 Table. Study characteristics and main measurement outcomes from studies modelling costing, cost-effectiveness, economic impacts by country.

|  | **LMICs^1^** | **India** | **South Africa** | **China** | **Indonesia** |
| --- | --- | --- | --- | --- | --- |
| **Nr. Studies** | 4 | 5 | 3 | 2 | 1 |
| **Vaccine type** | | | | | |
| BCG | 0 | 1 | 1 | 0 | 0 |
| M72/AS01E | 0 | 4 | 2 | 1 | 1 |
| Hypothetical vaccine^2^ | 4 | 2 | 0 | 1 | 0 |
| **Endpoint hypothetical vaccines^2^** | | | | | |
| PoI^3^ | 0 | 2 | 0 | 1 | 0 |
| PoD^4^ | 4 | 5=1 | 0 | 1 | 0 |
| PoR^5^ | 0 | 0 | 0 | 0 | 0 |
| **Target population** | | | | | |
| Adults and adolescents | 4 | 4 | 1 | 2 | 1 |
| Other | 0 | 1^6^ | 2^7^ | 0 | 0 |
| **Measurement outcomes** | | | | | |
| GDP^8^ gains | 1 | 0 | 0 | 0 | 0 |
| Cost-effectiveness | 2 | 2 | 2 | 1 | 0 |
| TB vaccination program cost | 0 | 3 | 0 | 1 | 0 |
| Threshold willingness to pay | 0 | 1 | 0 | 1 | 0 |
| Averted cost per income quintile | 1 | 0 | 0 | 0 | 0 |
| Full-income gains | 0 | 1 | 0 | 0 | 1 |

### 1.LMICs= Low-income countries (LIC), and lower and upper middle-income countries (LMIC). LICs are countries with a GNI per capita of $1,135 or less and LMICs with a GNI per capita between $1,136 and $4,465. Upper middle-income economies are those with a GNI per capita between $4,466 and $13,845. GNI per capita may differ per year ([World Bank Country and Lending Groups – World Bank Data Help Desk](https://datahelpdesk.worldbank.org/knowledgebase/articles/906519-world-bank-country-and-lending-groups)), 2.hypothetical vaccine= hypothetical vaccines often align with the WHO Preferred Product Characteristics (PPC)9 and do not adhere to the specific vaccine candidate characteristics currently in the pipeline, 3.PoI=prevention of infection, 4.PoD=prevention of disease, 5.PoR=prevention of recurrence, 6.Adolescents only, 7. Adolescents, PLHIV adults, 8.GDP=gross domestic product
