# Supplementary material for: Mapping the existing body of knowledge on new and repurposed TB vaccine implementation: A scoping review
Source: PLOS Glob Public Health. 2024 Aug 22;4(8):e0002885. doi: 10.1371/journal.pgph.0002885 (PMC11340902; doi:10.1371/journal.pgph.0002885)
Supplement: S5 Table — (DOCX) [file pgph.0002885.s006.docx]

S5 Table. Characteristics of studies evaluating facets related to implementation feasibility by country.

|  | India | South Africa | China |
| --- | --- | --- | --- |
| **Nr studies feasibility** | 2 | 4 | 1 |
| **Vaccine type** | | | |
| BCG | 1 | 2 | 1 |
| M72/AS01E | 1 | 2 | 1 |
| Hypothetical vaccine^1^ | 1 | 2 | 0 |
| **Endpoint hypothetical vaccines^1^** | | | |
| PoI^2^ | 0 | 1 | 0 |
| PoD^3^ | 1 | 2 | 0 |
| PoR^4^ | 0 | 0 | 0 |
| **Target population** | | | |
| Adolescents | 1 | 1 | 1 |
| Adults | 1 | 1 | 1 |
| Elderly | 1 | 0 | 1 |
| Socially vulnerable groups | 1 | 1 | 1 |
| PLHIV^5^ | 1 | 1 | 1 |
| Immunocompromised groups | 1 | 1 | 1 |
| Healthcare worker | 1 | 2 | 1 |
| Other | 2^6^ | 1^7^ | 1^8^ |
| **Implementation strategies** | 2 | 4 | 1 |
| **Health system readiness** | 1 | 1 | 1 |

1.hypothetical vaccine=hypothetical vaccines often align with the WHO Preferred Product Characteristics (PPC)9 and do not adhere to the specific vaccine candidate characteristics currently in the pipeline, 2.PoI=prevention of infection, 3.PoD=prevention of disease, 4.PoR=prevention of recurrence, 5.PLHIV= people living with HIV, 6= High-risk contact, high resource areas and occupational groups, spatially targeted vaccines in geographic hotspots, 7= Mining population, 8= High-risk contact, high resource areas and occupational groups.
