# Supplementary material for: Mapping the existing body of knowledge on new and repurposed TB vaccine implementation: A scoping review
Source: PLOS Glob Public Health. 2024 Aug 22;4(8):e0002885. doi: 10.1371/journal.pgph.0002885 (PMC11340902; doi:10.1371/journal.pgph.0002885)
Supplement: S6 Table — (DOCX) [file pgph.0002885.s007.docx]

S6 Table. Prioritized gaps for future research to guide new and repurposed TB vaccine implementation.

|  | **Geographical** | **Vaccine related** | **Data/ Tools** | **Other** |
| --- | --- | --- | --- | --- |
| **Overall** | Country specific evidence | - Other pipeline candidates - PoI^1^, PoR^2^ vaccines |  | - After 2050, i.e., long-term impact - Various target populations |
| **Epidemiological impact** | - Global, country specific evidence | - Other pipeline candidates - PoI^1^ vaccines, - Validated VE^3^ - Impact without prior BCG | - Country validated estimates. - Target population estimates | - Combination with other TB interventions - Country specific target populations |
| **Economic impact** | - Global, country-specific context | - PoD^4^, PoI^1^, PoR^2^ vaccines | - thresholds for cost-effectiveness | - Coverage - Timing of a program, - Combination with other TB intervention country specific target populations |
| **Implementation feasibility** | - Global, country specific evidence | - PoD^4^, PoI^1^, PoR^2^ vaccines | - Implementation strategies - Health system readiness assessment - Acceptability - Health equity | - Geographic analysis of accessibility to vaccine locations |
| **Acceptability** | - Global, country specific evidence | - PoD^4^, PoI^1^, PoR^2^ vaccines | - Attitudes, beliefs, perceptions, preferences, decision-making metrics | - Acceptability among target populations Preferences of target populations, including in comparison to other TB interventions |
| **Implementation strategies** | - Global, country specific evidence | - PoD^4^, PoI^1^, PoR^2^ vaccines | - Implementation strategies | - Country specific target populations |
| **Health system readiness** | - Global, country specific | - PoD^4^, PoI^1^, PoR^2^ vaccines | - Validated assessment tool - Country validated essential indicators | - Country specific target populations - Geographic analysis of catchment areas of expected vaccine locations |

1.PoI=prevention of infection, 2.PoR=prevention of recurrence, 3.VE= vaccine efficacy, 4.PoD
