# Supplementary material for: Mapping the existing body of knowledge on new and repurposed TB vaccine implementation: A scoping review
Source: PLOS Glob Public Health. 2024 Aug 22;4(8):e0002885. doi: 10.1371/journal.pgph.0002885 (PMC11340902; doi:10.1371/journal.pgph.0002885)
Supplement: Attachment — Submitted filename: Response to Reviewers.pdf [file pgph.0002885.s008.pdf]

**Rebuttal letter**

Dear editors of PLOS Global Public Health,

We kindly thank the reviewers for their feedback and the editors of the journal for the opportunity to submit a revised version of the manuscript. After careful review of the feedback, we have processed and incorporated most of the points raised by the reviewers. In this letter you can find our response to each comment, indicating how each point has been integrated into the revised version. We are also attaching the revised document with track changes and a clean version. We are positive that, with the adaptations made possible by the feedback of the reviewers, the manuscript has significantly improved and meets the journal's publication criteria.

Thank you in advance for considering this revised version.

Kind regards,

Joeri S. Buis

| Nr | Major comments | Response |
|---|---|---|
| 1 | I am a bit concerned about the language restriction. The finding that limited (published) data is available may well reflect that no articles in Spanish (Latin America), French (francophone Africa), Portuguese (Brazil), Russian (GAMTBVac is in phase 3) and Chinese (M. vaccae, in fact the one novel TB vaccine that is being implemented in health practice) were included. This is in particular important for studies looking at feasibility and acceptability. I would urge the authors to at least screen other language articles with English abstracts. | We thank the reviewer for raising this relevant point. Indeed some articles may have been missed due to the language restriction. We observed during the initial abstract-screening process of the English abstracts of non-English papers however, that the level of detailed information in the abstracts was limited. This would hinder assessment of compliance with the in- exclusion criteria without an additional full-text screening. Additionally, the abstracts are deemed to contain too limited information to extract for this review. Therefore, we have not executed an additional screening of non-English articles. We have discussed this point as a limitation in the discussion section and we encourage future similar reviews to include other non-English articles as well (lines 468-474). |
| 2 | Contrary to established practice there was no duplicate screening and article selection. Only a random sample was checked by the senior author. It would be important to know what proportion that sample was, and what exactly was checked. Only the abstracts, full texts? | We thank the reviewer for raising this relevant point. The removal of duplicates was performed manually during abstract-screening and full-text screening of the articles. We are positive this method was performed satisfactorily considering there are no duplicates included in the review. Additionally, 10% of the abstracts were screened by a senior author. There were no differences identified between the JSB screening and PTP 10% check. Consistency of the process was also upheld through regular meetings and discussion about article content. We have clarified the procedure in the method section (lines 195-202) |
| 3 | There is quite some attention to BCG revaccination, referring to a single trial with a prevention-of-infection endpoint. However, there have been at least two randomized trials that showed no effect of BCG revaccination on prevention of TB disease. This requires explanation in the | We thank the reviewer for raising this relevant point and we agree that the mixed result findings merit reflection in the discussion section, which we have included in the revised version (line 428-432). For the introduction we are merely highlighting the relevance of the review and the findings that have so far indicated the potential development of a new efficacious TB vaccine. Therefore, reflecting in the discussion section on the studies that found no effect was considered sufficient. |

| | | |
|---|---|---|
| | introduction section and reflection in the discussion section. | |
| | Minor comments | |
| 4 | Tables don't seem to number through. | We thank the reviewer for the feedback. We have adjusted this by numbering the tables in the text and separately numbering the tables in the supplementary material. In the revised version, tables in the supplementary material are referred to as (see supplementary table, S1. An example can be found in line 259-260. |
| 5 | Abstract 41 adult | We thank the reviewer for the feedback. This has been adjusted in the text, line 41 |
| 6 | 91 Reference 3 is unpublished and not accessible. Please provide alternative(s). | We thank the reviewer for the feedback. This has been adjusted in the text, line 91. Ref: **Infant BCG vaccination and risk of pulmonary and extrapulmonary tuberculosis throughout the life course: a systematic review and individual participant data meta-analysis - The Lancet Global Health** |
| 7 | 98-99 These include both vaccines for preventive indication and vaccines for therapeutic indication (MIP). It would seem important to make that distinction. In addition: there is no mention of M. vaccae. | We thank the reviewer for the feedback. We rephrased the sentences and focused on the vaccines with POI and/or POD. We relied on the pipeline report of TAG 2023 as a reference. The m.vaccae vaccine is not considered a vaccine in clinical development in this reference. |
| 8 | 107 Please define "repurposed". | We thank the reviewer for the feedback. The sentence has been adjusted to clarify "The BCG revaccination is being repurposed for adults and adolescents and efficacy was assessed, in adolescents between the ages of 12-17 years", line 102-104 |
| 9 | 114 Please explain why excluding TB infection is relevant. | We thank the reviewer for the feedback and the text has been clarified into "Harris et al. argue that some approaches used in the modelling – such as campaigns specifically targeting uninfected people which is necessary for PoI vaccines – may not be feasible due to the need to screen for TB infection prior to vaccination which will include high cost and inaccuracies of available tuberculin skin tests to exclude TB infection8."(line 115). |
| 10 | 150 Was there a protocol? Is that available? Was the review registered? | We thank the reviewer for the feedback. There was a protocol reviewed and approved by independent internal review panel before the start of the review. However, the review was not publicly available on for example, Prospero. We uploaded a copy of the approved protocol as supplementary information. |
| 11 | 168 Who are meant by "the internal team"? | We thank the reviewer for the feedback. We refer to the team that has worked on this product namely, the authors. We have clarified this in the text. This has also been clarified in line 159. |
| 12 | 176 "moving to or that are" | We thank the reviewer for the feedback. Adjusted in text and now reads "Studies focusing on vaccine candidates moving to or that are in phase 3 trials were included as were hypothetical vaccines". Line 179-180. |
| 13 | 177 Important to define "generic". Consider calling it "hypothetical" instead. | We thank the reviewer for the feedback. We have adjusted the text and changed generic to hypothetical. We included a short explanation of a hypothetical vaccine in the method section "Hypothetical vaccines often align with the WHO Preferred Product Characteristics (PPC) and do not adhere to the specific vaccine candidate characteristics currently in the pipeline", line 180-182. |
| 14 | 189 Provide reference for Zotero. | We thank the reviewer for the feedback. This has been added in the text, line 193, reference 12. |
| 15 | 197 "thorough" - please clarify. | We thank the reviewer for the feedback. We removed this word to increase readability for the reader. With thorough we meant that the checking and |

| | | |
|---|---|---|
| | | cross-checking of references were taken serious and particular attention was given to this process to avoid overlooking any relevant articles. |
| 16 | 204 AIGHD/EDCTP - is that the same as the roadmap mentioned earlier? | We thank the reviewer for the feedback and yes it is the same roadmap. We have clarified this in the text, line 209 and 119. |
| 17 | 208 missing ")" | We thank the reviewer for the feedback. This has been added in the text, see line 214 |
| 18 | 211 "the potential" | We thank the reviewer for the feedback. This has been added in the text, see line 115 |
| 19 | Was any quality appraisal of the included articles conducted? | We thank the reviewer for the feedback. We conducted a scoping review which most commonly does not include a formal assessment of quality as mentioned by Grant and Booth (A typology of reviews: an analysis of 14 review types and associated methodologies - PubMed (nih.gov) and the PRISMA guidelines for Scoping reviews (PRISMA Extension for Scoping Reviews (PRISMA-ScR): Checklist and Explanation - PubMed (nih.gov)). Our objective and scope were to map and synthesize the existing knowledge rather than evaluating quality or risk of bias in the results Future research could however, assess the best quality evidence using different types of reviews and including a quality assessment of papers. |
| 20 | 240 Please explain "readiness focus" and "vaccine endpoints". | We thank the reviewer for the feedback. This has been clarified in the method and result section. See line 212, 222, 247. |
| 21 | 265 I don't think these studies were done in these countries but for these countries. | We thank the reviewer for the feedback. This has been adjusted in the text, see line 272. |
| | 285 "Mycobacterium" (italicized); recovered from...? | We thank the reviewer for the feedback. We italicized mycobacterium and added "from TB". See line 298<br><br>"protective in both *mycobacterium* tuberculosis (Mtb.) infected and recovered from TB populations" |
| | 291 "proportion" instead of "concentration" | We thank the reviewer for the feedback. This has been adjusted in the text. See line 304 |
| | 294 what is meant by "well-connected"? | We thank the reviewer for the feedback. Well-connected refers to an area with high interaction/contact between the people. The sentence has been adjusted to "Vaccination of people in ahigh TB incidence area with high population density and movement, could reduce incidence 1.6-fold or more". See line 307-308. |
| | 315 "cost" instead of "costing" | We thank the reviewer for the feedback. This has been adjusted in the text |
| | 319 please clarify "due to the epidemic" | We thank the reviewer for the feedback. This has been adjusted in the text, see line 320-322. "Additionally, Harris et al. found that only a vaccine which includes efficacy in post- & pre-infected people was cost-effective in India because, the TB epidemic is equally driven by new infections of susceptible people as re-infection of people with TB infection (TBI)and due to their level of thresholds for cost-effectiveness. |
| | 325 please clarify "RR-TB" | We thank the reviewer for the feedback. This has been adjusted and clarified in the text, see line 328. |
| | 315-327 This observation is difficult to interpret unless the impact of the two approaches was similar. Was it? | We thank the reviewer for the feedback. Yes, the impact of the approaches was similar. This has been clarified in the text, see line 328-330. |

| | | |
|---|---|---|
| | | "Another study by Harris et al. found that in India and South Africa, the costs of vaccinating 18 year olds with 50% coverage was equal to the costs of vaccinating 15 year olds with 80% coverage, and these two approaches had a similar impact" |
| | 332 Leave out table title. | We thank the reviewer for the feedback. This has been removed in the text. See line 332. |
| | 348 part of sentence is missing | We thank the reviewer for the feedback. The missing part of the sentence has been added. See line 348 |
| | 352 "All five" | We thank the reviewer for the feedback. The suggestion has been added in the text. See line 352 |
| | 365 Leave out table title. | We thank the reviewer for the feedback. This has been removed in the text. See line 365 |
| | 368 What are "repurposed cold chain requirements"? | This has been an editing mistake. Thank you for pointing this out. It has been removed in the text, line 368 and in table 11. |
| | 379 "potential" | We thank the reviewer for the feedback. Adjusted in the text. See line 379 |
| | 381 "followed by" - stylistically awkward sentence | We thank the reviewer for the feedback. Adjusted in the text. See line 382<br><br>"Most studies assessed epidemiological or costing/cost-effectiveness and/or economic impacts of new and/or repurposed TB vaccines. Limited research looked at implementation feasibility and only one study contained information regarding acceptability of new and repurposed TB vaccines among high level stakeholders". |
| | 408-409 please clarify "prior research confirms the few findings" | We thank the reviewer for the feedback. Prior research refers to research that has looked at the implementation of adult vaccines other than new and or repurposed TB vaccines. It has been clarified in the text in line 410 and 414. |
| | 410-411 "monitoring and surveillance systems" - of what? | We thank the reviewer for the feedback. This has been specified in the text. We specifically refer to monitoring systems to keep track of side effects, see line 413. |
| | 412 "need for estimating" | We thank the reviewer for the feedback. This has been added to the text. Line 414. |
| | 427-428 Please add this is only relevant if these vaccines are proved effective in regulatory trials. | We thank the reviewer for the feedback. This has been added to the text, see line 428-432.<br><br>"Thirdly, while BCG revaccination and M72/AS01E vaccine candidates are advanced, they may not be suitable for all contexts and populations if proven effective in regulatory trials. Several vaccines (M72/AS01e and MTBVAC) are entering late stage trials and there is still a need for proven effectiveness in regulatory trials. For example, previous studies found no effect of the BCG revaccination against the development of TB infection or disease[45–47]. Therefore, investments in and development of new TB vaccine candidates will remain crucial.." |
| | 430 "approvable" - by whom? | We thank the reviewer for the feedback. The sentence has been changed to : "Further clinical research outcomes of vaccine candidates in the pipeline will guide the parameters to consider in future modelling". See line 434 |
| | 432 "opinion"; why "but"? | We thank the reviewer for the feedback. It has been changed to "and", see line 436 |
| | 433 please clarify "necessary" | We thank the reviewer for the feedback. The word "necessary" has been deleted, see line 437 |
| | 435-436 "other (...) candidates" | We thank the reviewer for the feedback. The word other has been added |

| | | |
|---|---|---|
| | 439 "paramount to" | We thank the reviewer for the feedback. “Paramount for” has been changed into “paramount to”, see line 443. |
| | 453 why "subsequently"? | We thank the reviewer for the feedback. The word “subsequently” has been deleted, see line 457. |
| | 454 please clarify "conspicuous" | We thank the reviewer for the feedback. The word “conspicuous” has been changed into “large”, see line 459 |
| | 457-459 But there is an extensive literature on this topic for non-TB vaccines. | We thank the reviewer for the feedback. We agree that extensive research has been done on non-TB vaccines. We added a sentence that mentions that future research should make use of this already existing knowledge base, see line 461-464.<br><br>“The absence of research in this domain underscores the urgent necessity for comprehensive investigations and leveraging insights from existing research on non-TB vaccines aimed at better comprehending the dynamics that shape individuals’ attitudes, perceptions, and behaviours towards a potential new TB vaccine”. |
| | 461 leave out "(" | We thank the reviewer for the feedback. “(“ has been deleted. |
| | 470 why "inadvertently"? It suggests the screening was not done too thoroughly. | We thank the reviewer for the feedback. The search has been done thoroughly and we conducted it using multiple methods, increasing our potential yield to find relevant literature. However, it may still be that some studies -unknown by the authors- have not been published in the search engines or PLOS site and are therefore, not identified. We deleted the word inadvertently to avoid this association with a non-thorough search, see line 471. |
| | 474 "hepatitis" | We thank the reviewer for the feedback. The capital H has been turned into a small h, see line 476 |
| | Table 1<br>Children is defined up to what age (to separate them from adolescents) | We thank the reviewer for the feedback. Children are younger than 9 years of age. This has been added to table 1. |
| | Figure 2<br>Provide reasons for exclusion (grouped) | We thank the reviewer for the feedback. The exclusion criteria have been added to the figure. |
| | Table 4<br>Is there any information on the duration of vaccine protection assumed in these studies? | We thank the reviewer for this comment and yes there is information on duration of protection. This has been added in the tables. Because this was perceived a significant change, we have also referenced it in the results section (line 290) and in the discussion (line 397). Table 5 has also been updated with duration of protection. |
| | What is the rationale for the order of articles? It seems neither alphabetical not chronological. | We thank the reviewer for this comment. This has been adjusted and the tables are ordered according to the number of countries included in the studies; with the highest number of countries at the top and single countries at the bottom. There are multiple single countries so therefore the order for the single countries is alphabetical based on country name. The studies focusing on the same country are alphabetically ordered according to the authors. |
| | Please provide clarification of all abbreviations. | We thank the reviewer for pointing this out. Abbreviations have been added to the bottom of all the tables in the text. |
| | Please be clear and consistent about what the numerical intervals represent. What does "range" mean in this regard? | This has been checked, adjusted, and clarified in table 4 |
| | Please clarify what is meant by "POI+POD", "pre-infection and post-infection" | We thank the reviewer for the feedback. This clarifications have been added to the table 4 at the bottom. |

| | | |
|---|---|---|
| | | pre-infection and post-infection vaccine= a vaccine effective in people regardless of infection status<br><br>POI+POD has been adjusted to vaccine with POI & POD. Clarification of POI and POD are added to the bottom of table 4 |
| | Liu 2017: please clarify "pulse" | We thank the reviewer for the feedback. This has been changed to "periodic" in table 4. |
| | Fu 2014: "interval" | We thank the reviewer for the feedback. This has been changed to CrI and at the bottom of the table clarified as credible intervals as was abbreviated in the article |
| | Shrestha 2017: what are "repurposed miners"? | We thank the reviewer for the feedback. This is a typo and has been removed. |
| | Harris 2019: what do the intervals refer to? | We thank the reviewer for the feedback. This has been checked and in the article they refer to uncertainty intervals. This has been adjusted in the table. |
| | Awad 2020: please clarify "conservative" | We thank the reviewer for the feedback. This has been clarified at the bottom of table 4.<br><br>Conservative= duration of protection is 10 years and no reduction in infectiousness of those with TB disease nor a reduction in progression rate to TB disease for those who are slow progressors |
| | Shrestha 2016: is this incidence reduction population-wide or in the hotspots only? | We thank the reviewer for the feedback. It is in a reduction population wide (the study was modelled for the state of Gujarat) and not specific to the hotspots. This has been clarified in table 4. Thank you for bringing this up. |
| | Renardy 2019: "Yearly..." suggests that every year the same reduction is predicted. I don't think that is what is meant. | We thank the reviewer for the feedback. We have removed the word yearly, it is indeed confusing and not necessary in the context |
| | See general comments Table 4 | We thank the reviewer for the feedback. The same comments for table 4 have been incorporated into table 5. |
| | Weerasuriya 2021: WTP thresholds are generally for cost/DALY. Here they seem to refer to a total amount. Please clarify. | We thank the reviewer for the feedback. We have added the specific cost/DALY. |
| | Harris 2022: Please provide an interpretation of the negative cost/DALY estimates | We thank the reviewer for the feedback. These negative costs indicate that the scenario is cost-saving. This has been added to table 5. |
| | Due 2013: What does the range of cost/DALY represent? | We thank the reviewer for the feedback. This has been clarified in table 5. The range is dependent on the vaccine price and the VE. They modelled with a vaccine price between \$1-\$10 and a VE between 10%-80%. |
| | Jayawardana 2022: Please clarify "total incremental cost" | We thank the reviewer for the feedback. We used the way it was formulated in the article itself . "The total incremental cost of the 2xMass18–50 strategy compared to no vaccination was USD 417 million (IQR 400–433 million)". They refer to the incremental costs of the entire vaccination program. This has been clarified in the table. |
| | See general comments Table 4 | We thank the reviewer for the feedback. Abbreviations have been added under the table |
| | Table 10 I assume "not specific" should read "not specified" | Correct, we thank the reviewer for the feedback. It has been adjusted in the table. |
| | Shrestha 2017: if not specified, why is there a timespan? | We thank the reviewer for the feedback. The "not specified" has been removed. |

| | | |
|---|---|---|
| | Table 11 See general comments Table 4 | We thank the reviewer for the feedback. The abbreviations have been added under the table. |
| | HIS acceptability: I find this barrier surprising. All three countries have established pharmacovigilance systems. So, what exactly is meant here? | We thank the reviewer for the question. In the original article it is a point raised by the participants in all three countries to either have a proper system set up for monitoring and surveillance of adverse events or have proper training. It is not so much to create an established pharmacovigilance as to strengthen it. This nuance has been incorporated in the table. |
| | References<br>37 is duplicated as 42 | We thank the reviewer for the feedback. The references to this article are all reference nr 39 now. |
