# Supplementary material for: Mapping the existing body of knowledge on new and repurposed TB vaccine implementation: A scoping review
Source: PLOS Glob Public Health. 2024 Aug 22;4(8):e0002885. doi: 10.1371/journal.pgph.0002885 (PMC11340902; doi:10.1371/journal.pgph.0002885)
Supplement: Attachment — Submitted filename: Response to Reviewers_June24.docx [file pgph.0002885.s009.docx]

**Rebuttal letter**

Dear editors of PLOS Global Public Health,

We kindly thank the reviewer for their feedback and the editors of the journal for the opportunity to submit a new revised version of the manuscript. We have processed and incorporated all points raised. In this letter you can find our response to each comment, indicating how each point has been integrated into the revised version. We have attached the revised document with track changes and a clean version. We are hopeful that, with the adaptations made possible by the feedback of the reviewers, the manuscript meets the journal’s publication criteria.

Thank you in advance for considering this revised version.

Kind regards,

Joeri S. Buis

| Nr | Minor comments | Response |
| --- | --- | --- |
| 1 | 98-99: Why "vaccines against prevention..."? I assume vaccines ae meant to prevent. The sentence furthermore suggests that some of these vaccines are meant primarily as sterilizing vaccines (POI). I don't think that is the case. Some vaccines/candidates have been or are being evaluated against a POI endpoint, but that's something different. | We thank the reviewer for the comment. We understand that the vaccine may be evaluated against a PoI endpoint, which may differ from the final outcome of protection of the vaccine. We have therefore adjusted the sentence to: *These six vaccines—MIP, VMP1002, GamTBvac, MTBVAC, BCG (re)vaccination, and M72/AS01E—are being tested for their potential to prevent disease (PoD) or prevent infection (PoI).* See line 98-99 in the clean revised version. |
| 2 | 105: Is this indeed the statement by Harris at al? The concerns about pre-vaccination testing to my knowledge are about vaccines that have only been licensed for use in IGRA-positive individuals, that's not POI. | We thank the reviewer for the comment. Harris et al. argues the following in their discussion section:  *Upon implementation, sustained campaigns speciﬁcally targeting either uninfected (with a pre-infection vaccine) or latently infected populations (with a post-infection vaccine) may not be feasible. To identify such populations, TST or IGRA testing would be required. Such tests come with cost and organizational implications, and neither are perfect tests for latency or the absence of infection.*  We have adjusted the sentence to take the nuance of the reviewer into account and to align more clearly with the statement by Harris et al. Line 113-118 in the cleaned revised version:  *"However, Harris et al. argue that the assumptions used in the modelling—such as having separate vaccines specifically licensed for people who are not yet infected (pre-infection vaccine) or have been infected (post-infection vaccine)—may not be feasible. This infeasibility arises from the need to screen for TB infection prior to vaccination, which incurs high costs and relies on tuberculin skin tests or IGRA tests. These tests are not only expensive but also have limitations in accurately diagnosing TB infection status, thus complicating targeted vaccination efforts."* |
| 3 | 199-200: my comment was not about duplicate articles about screening by two independent reviewers. I still believe the manuscript should state the percentage discrepancy in the 10% random sample so that the reader can be convinced that single review of the remaining 90% did not lead to major bias. | We thank the reviewer for their comment and clarification. The 10% check by the second reviewer did not lead to any changes in the selection of articles. We have clarified this in the text, line 197-202 in the revised cleaned version.  *“JSB conducted the initial selection, screening, and assessment of articles. Weekly meetings were held to discuss any articles for which JSB was uncertain about their inclusion in the review. These meetings further specified the inclusion process where needed. Additionally, PTP reviewed a random sample comprising 10% of the articles to ensure consistency in the inclusion process. The verification performed by PTP did not necessitate any changes to the selection of the articles.”* |
